# Over-expression of mammaglobin-B in canine mammary tumors

**DOI:** 10.1186/s12917-018-1507-z

**Published:** 2018-06-15

**Authors:** Mamta Pandey, B. V. Sunil Kumar, Kuldip Gupta, Ram Saran Sethi, Ashwani Kumar, Ramneek Verma

**Affiliations:** 1 School of Animal Biotechnology, Guru Angad Dev Veterinary and Animal Sciences University, Ludhiana, Punjab 141004 India; 2 Department of Veterinary Pathology, Guru Angad Dev Veterinary and Animal Sciences University, Ludhiana, 141004 India; 3 Department of Veterinary Surgery and Radiology, Guru Angad Dev Veterinary and Animal Sciences University, Ludhiana, 141004 India

**Keywords:** Canine, Expression, Immunohistochemistry, Mammaglobin-B, Mammary tumor, qRT-PCR

## Abstract

**Background:**

Mammaglobin, a member of secretoglobin family has been recognized as a breast cancer associated protein. Though the exact function of the protein is not fully known, its expression has been reported to be upregulated in human breast cancer.We focused on studying the expression of mammaglobin-B gene and protein in canine mammary tumor (CMT) tissue. Expression of mammaglobin-B mRNA and protein were assessed by quantitative real-time polymerase chain reaction (qRT-PCR) and immunohistochemistry (IHC), respectively.

**Results:**

High levels of mammaglobin-B mRNA expression (6.663 ± 0.841times) was observed in CMT as compared to age and breed matched healthy controls. Further, expression of mammaglobin-B protein was detected in paraffin-embedded mammary tumor tissues from the same subjects by IHC. Mammaglobin-B protein was overexpressed only in 6.67% of healthy mammary glands while, a high level of its expression was scored in 76.7% of the CMT subjects. Moreover, no significant differences in terms of IHC score and qRT-PCR score with respect to CMT histotypes or tumor grades were observed, indicating that mammaglobin-B over-expression occurred irrespective of CMT types or grades.

**Conclusion:**

Overall, significantly increased expression of mammaglobin-B protein was found in CMTs with respect to healthy mammary glands, which positively correlates to its transcript. These findings suggest that overexpression of mammaglobin-B is associated with tumors of canine mammary glands.

## Background

Mammary tumors are the second most common tumor type, surmounted by skin tumors in dogs primarily affecting unspayed bitches [1]. Early diagnosis of the disease is a challenging task in animals especially in countries like India where periodic visit of dog owners to a veterinarian is not a regular practice. Sometimes, the size of tumor might be very small and located deep within the body making early diagnosis a difficult task. Moreover, the signs and symptoms like pain, weight loss, weakness, etc. are not tumor specific. Few subjects may not at all show any signs and symptoms until the cancer has reached its advanced stages. Hence, it is diagnosed at a late end point of carcinogenesis. Further, in later stages there are more chances of tumor metastasis, leaving the treatment module ineffective [1]. A higher incidence of mammary tumors in canines and the need to diagnose them at an early stage are motivating factors to aim development of better screening and diagnostic methodologies. In this pursuit, discovery of novel biomarkers, which are sensitive and specific enough to diagnose this malady, are essentially required.

Mammaglobin-B is one of the 23 members of the secretoglobin superfamily, a group of small, secretive, seldom glycosylated proteins [2]. Gene encoding mammaglobin-B was identified in the studies of differentially expressed cDNAs from carcinoma cell lines of human breast cancer [3]. Unlike other members, its expression is highly specific to the mammary gland and is expressed at a low level in normal and healthy mammary tissue [4]. It is detected in breast tissue as a complex with lipophilin B [5] and its expression is reported to increase with breast carcinogenesis [4]. Increased expressions of mammaglobin have been observed in breast cancer derived cell lines [6] and human breast cancer tissues [3, 7–12]. Although its role in cancer immunopathology is not clearly defined, but it is thought to be involved in signalling, immune response, chemotaxis and are possibly carriers of steroid hormones [13]. It is also considered as a promising molecular marker for disseminating and circulating breast cancer cells [4]. So far, the mammaglobin expression studies are well reported for human breast cancers but no such reports are documented for mammary tumors in canines. In our previous study we reported detection of this protein for the first time in the serum of canines with mammary carcinomas [14].

Quantitative real time-PCR (qRT-PCR) and immunohistochemical diagnosis of canine mammary tumors (CMTs) targeting tumor specific markers are the most widely used methods to score the expression status of markers. This will lead to introduction of new biomarkers, which may aid in treatment and prognosis of the tumorous condition. In addition, diagnosis could be done at an early stage that may increase the survival period of afflicted dogs. In this context, the purpose of this study is to quantify mammaglobin-B transcript by qRT-PCR and protein by immunohistochemical analysis in CMTs. In this study, for the first time we presented the expression of mammaglobin-B at mRNA and protein levels and their correlation with various histotypes of CMTs and their histological grades of malignancy.

## Methods

### Animals and tissue samples

Biopsies from tumor free apparently healthy mammary glands (*n* = 30) and mammary tumor tissues (*n* = 30) were collected from age and breed matched female dogs brought to Department of Veterinary Surgery and Radiology of Guru Angad Dev Veterinary and Animal Sciences University, India. After the removal of tumors and post-operative care, the animals were discharged from the clinics and allowed to go with their owners. All the animals under investigation were in the age group of 5–12 years (Median: 7.25). The animals had neither received any treatment even in the form of any non-steroidal anti-inflammatory drug nor chemotherapy prior to surgery. Only unsprayed female canine subjects were considered for the present study. Healthy canine mammary gland biopsies were also taken from the subjects that were brought to the clinics. The samples were stored in RNA*later* (Sigma, USA) as well as 10% neutral buffered formalin (NBF) for RNA isolation and immunohistochemistry (IHC), respectively. Permission for collection of the samples from the canines used in this experiment was approved by the institution’s animal ethics committee vide edorsement no. VMC/12/3901–35.

### RNA isolation and complementary DNA (cDNA) synthesis

Total RNA was extracted from the tissue samples using TRIzol (Thermo Scientific, USA) [15]. 1 μg of isolated RNA from each sample was reverse transcribed to cDNA using Oligo dT primers and RevertAid premium first strand cDNA synthesis kit (Thermo Scientific, USA) following the manufacturer’s instructions.

### Quantitative real-time PCR (qRT-PCR)

cDNA isolated from healthy and tumorous mammary tissues were used for qRT-PCR. Using the available sequence of canine mammaglobin-B (AB971219.1) from NCBI, gene specific oligonucleotide primers (GCAATGTTTTCTCCAGCAGTCG and GTCCCTGTCCACTGGTTTGAAA) were designed for qRT-PCR. Beta actin (β-actin)gene with primer sequences: CCGCGAGAAGATGACCCAGA and GTGAGGATCTTCATGAGGTAGTCGG [16] and RPS19 gene with primer sequences: CCTTCCTCAAAAAGTCTGGG and GTTCTCATCGTAGGGAGCAAG [17] were used as valid internal controls*.* RPS19 gene encodes for ribosomal protein S19 of the 40S ribosomal subunit. This gene had been used as valid internal control in earlier cancer studies [18]. Non template control was kept to check for non-specific amplification. All the primers were used at 0.2 μM final concentration. Power SYBR Green QPCR master mix (Applied Biosystems) was used for amplification of the genes. Samples were run in triplicates. The dissociation curves were generated between 60 and 95 °C to assess specificity of amplicons. The threshold cycle (Ct) values obtained for each test and internal control genes (β-actin and RPS19) after 40 cycles of amplification were used to measure the relative expression of mammaglobin-B gene in tumor and healthy tissues as per Abasht et al. (2009) [19]*.* Statistical analyses were done as per Livak and Schmittgen (2001) [20] and comparisons were made by student’s t test using SAS version 9.3. The percent PCR amplification efficiencies (E) for each of the assay was calculated as E = (10^–1/slope^ - 1) × 100 [21].

### Histopathology

About 5 μm sections of paraffin wax embedded tumor tissue samples were stained with haematoxylin and eosin (HE) for microscopic evaluation. All the tumors were classified according to World Health Organization (WHO) criteria of tumor classification [22] with slight modifications as per Goldschmidt et al. (2011) [23]. Thirty CMTs were classified into 13 complex carcinomas (43.3%), 11 simple carcinomas (36.7%), 4 carcinosarcomas (13.3%) and 2 fibrosarcomas (6.7%). The histological grades of malignancy of the CMTs were also assessed according to Goldschmidt et al. (2011) [23]. About 33.33% tumors (*n* = 10) were found to be of grade I (well differentiated), 60% (*n* = 18) of grade II (moderately differentiated) and 6.67% (*n* = 2) of grade III (poorly differentiated).

### Immunohistochemistry

Previously we have cloned and heterologously expressed canine mammaglobin-B gene in *E. coli* as 12 kDa recombinant fusion protein and raised hyperimmune sera against the expressed protein in rabbits [14]. IgG purified from the hyperimmune sera [14] was used as primary antibody for IHC. Total proteins were extracted from the mammary tumor tissues [24] and Western blot [25] was carried out using the purified IgG to assess specificity of the raised antibodies.

The IHC technique was employed as standardized earlier [26] with slight modifications. Briefly, 3 μm-thick sections were cut from the tissue embedded blocks and fixed to poly- L-lysine-coated slides. The slides were de-paraffinized and rehydrated in graded alcohols. A modification of heat-induced epitope retrieval was done by boiling the sections first in citrate buffer at 70 W for 15 min followed by a brief wash with deionized water and boiling the sections in tris-ethylene diamine tetra acetic acid (EDTA) buffer at 70 W for 10 min in a microwave oven. Sections were then allowed to cool down to room temperature in tris-EDTA buffer. Endogenous peroxidase was quenched by incubating sections in 3% hydrogen peroxide solution in methanol for 20 min. To reduce non-specific binding, slides were incubated with power block (Vector Laboratories, USA) for 15 min at room temperature. Primary antibody was used at 1:200 dilution, and slides were incubated overnight in a humidified chamber at 4 °C. IgG purified from pre-immune sera was used as negative control to confirm the specificity of immunostaining [27]. Horseradish peroxidase (HRPO) conjugated goat anti-rabbit IgG (Santa-Cruz Biotechnology, USA) was used as secondary antibody at a dilution of 1:2500. A freshly prepared 3, 3′-diaminobenzidine (DAB) solution (DAB Peroxidase Substrate Kit-Vector Laboratories, USA) was used to visualise the colored reaction. Finally, the slides were immersed in distilled water, counterstained with Gill’s haematoxylin (Sigma GH5–2-16) stain and permanently mounted.

### Semiquantitative scoring of mammaglobin positive cells

The immunohistochemical analysis of positive cases was done by semiquantitative method in which both the intensity of the brown color developed and the percentage of cells showing positive staining were taken into consideration. The scoring was done by pathologists blinded to patient’s history [28]. To calculate the staining index (SI or H-score), the score allotted for the percentage of immuno-positive cells was multiplied by the score allotted for the staining intensity.The value calculated for SI score was used to define higher (SI ≥ 6) or lower (SI < 6) expression of the mammaglobin-B protein at tissue level [28, 29]. The percentage of immuno-positive cells was obtained from 20 random fields per case per section using a 40X objective lens. The percentage of positive cells, was quantified by arbitrarily assigning scores as follows: 0–25% positive cells: 1; 26–50% positive cells: 2; 51–75% positive cells: 3; 76–100% positive cells: 4. Staining intensity i.e. severity of brown color developed after staining was scored as 0 in case of no staining, 1 for weak staining, 2 for moderate staining and 3 for strong staining.

### Statistical analysis

All statistical analyses were performed using statistical analysis software (SAS ver. 9.3). The association between H-score and 2^−ΔCT^ (qRT-PCR score) was determined using the Pearson correlation coefficient (r). Spearman’s rank correlation between the scores was also calculated to assess how well the relationship between the two variables could be described using a monotonic function. Scatter-plots were generated for graphical illustrations of correlation between H- and qRT-PCR-scores. Regression analysis was also performed for exploring the relationship between H-score and qRT-PCR score. The Kruscal Wallis test was used to determine statistical association of H scores, qRT-PCR score and grades of tumor with the tumor histotypes.

## Results

### Mammaglobin mRNA expression

**Fig. 1:**
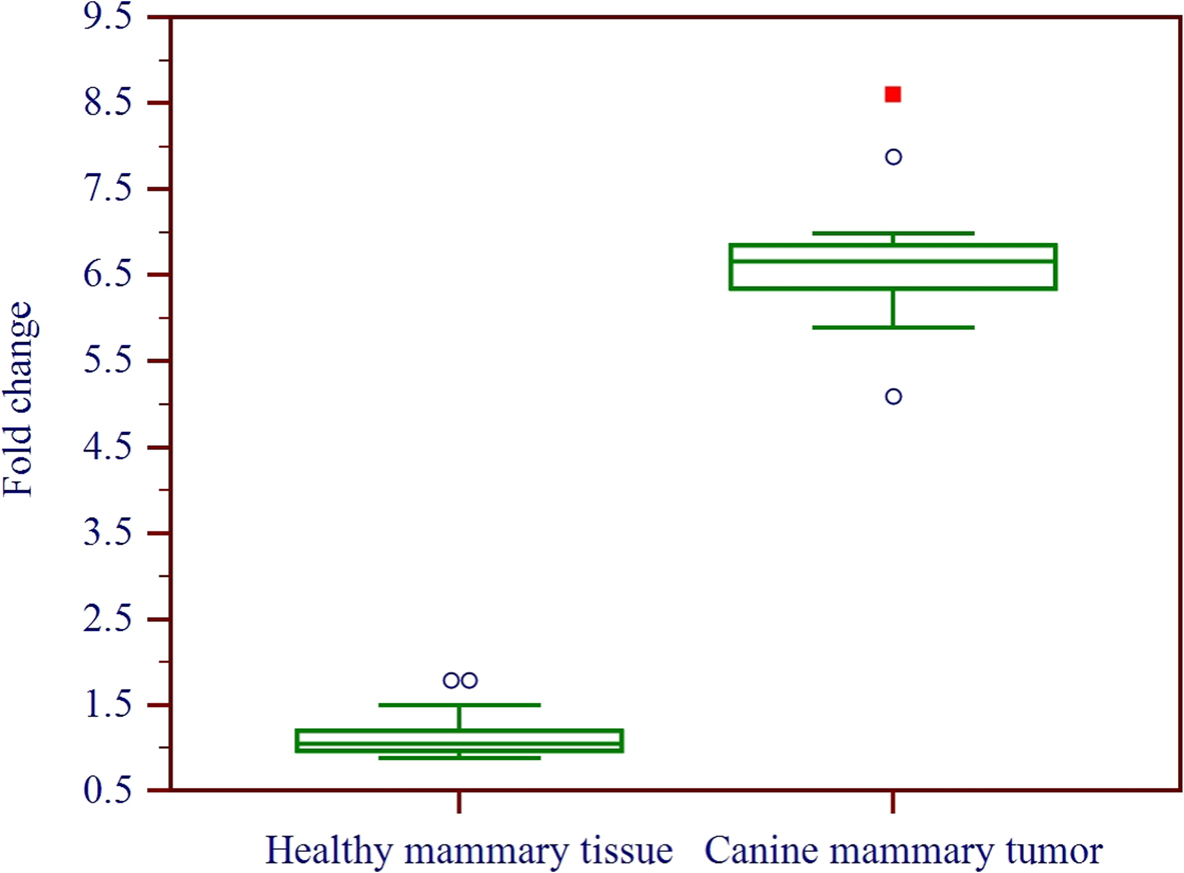
Box and whisker’s plot showing fold change in mammaglobin-B gene expression in healthy and tumorous mammary tissue

Amplification efficiencies of mammaglobin-B, β-actin, and RPS19 genes were found to be 91.6, 93.8, and 99.4% respectively, indicating appropriate exponential efficiencies for these reactions. Mammaglobin-B gene was found to be 6.663 ± 0.841 times significantly up-regulated (*P* ≤ 0.05) in CMT as compared to the healthy glands (Fig.1).

### Mammaglobin protein expression

**Fig. 2:**
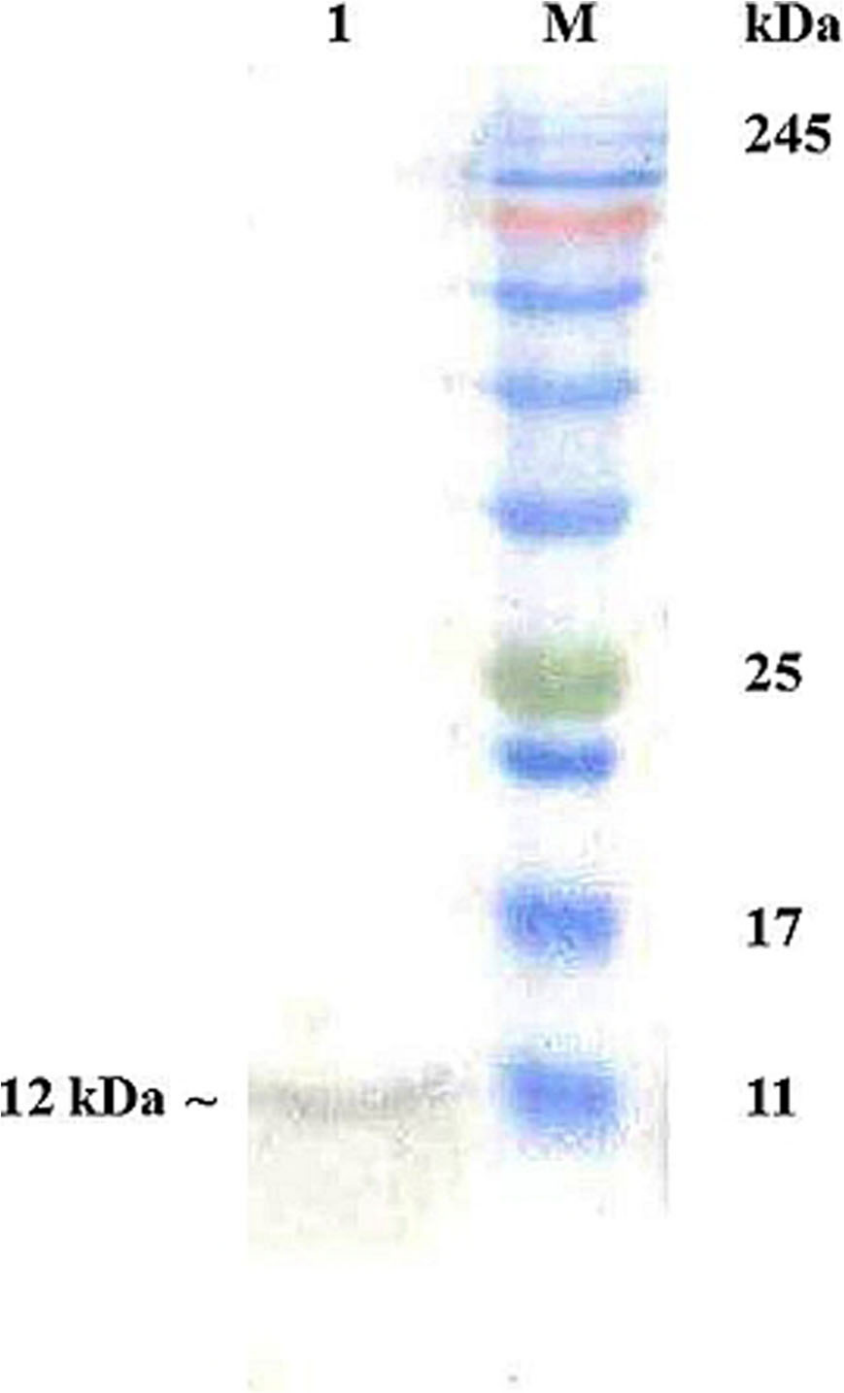
Western Blotting for checking specificity of purified IgG against mammaglobin-B protein (Lane M: Pre-stained protein ladder; Lane 1: immunoreactive mammaglobin-B protein in CMT tissue)

IgG purified from the hyperimmune serum reacted specifically with mammaglobin–B on nitrocellulose membrane out of the total proteins extracted from tumor tissue (Fig.2), confirming specificity of the purified IgG against canine mammaglobin-B.

**Fig. 3:**
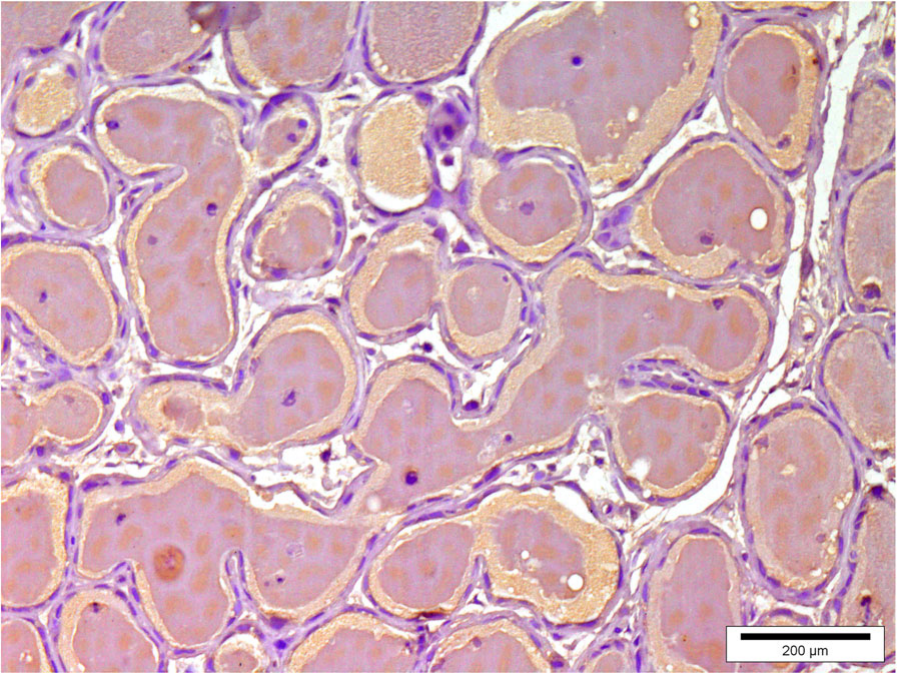
Immunohistochemistry of healthy canine mammary grand depicting low levels of mammaglobin-B immunopositive cells. Bar =200 μm

**Fig. 4:**
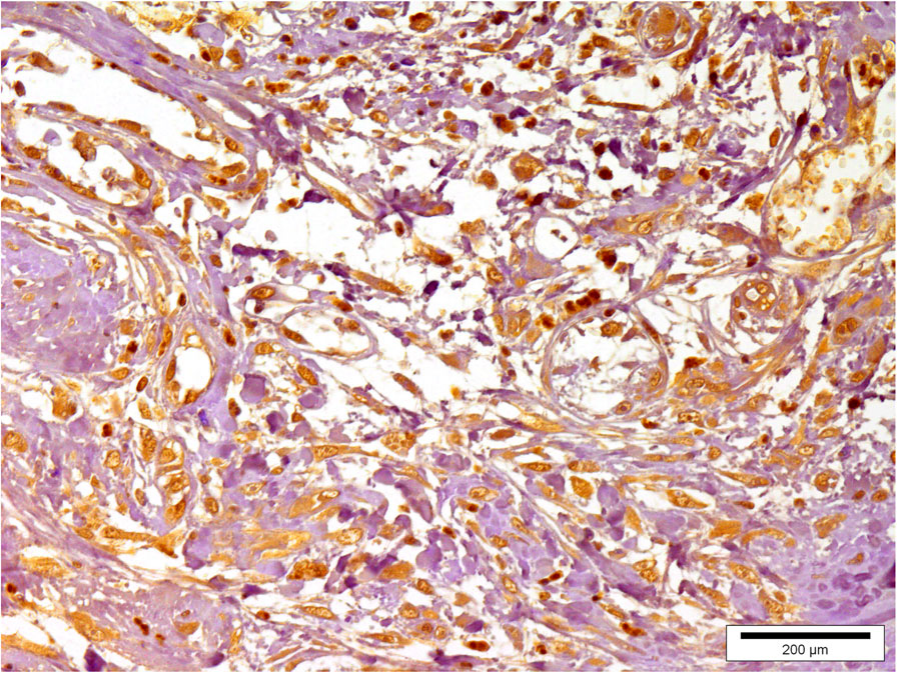
Immunohistochemistry of canine mammary tumor (Complex carcinoma showing mammaglobin-B immunopositive cells). Bar = 200 μm

**Fig. 5:**
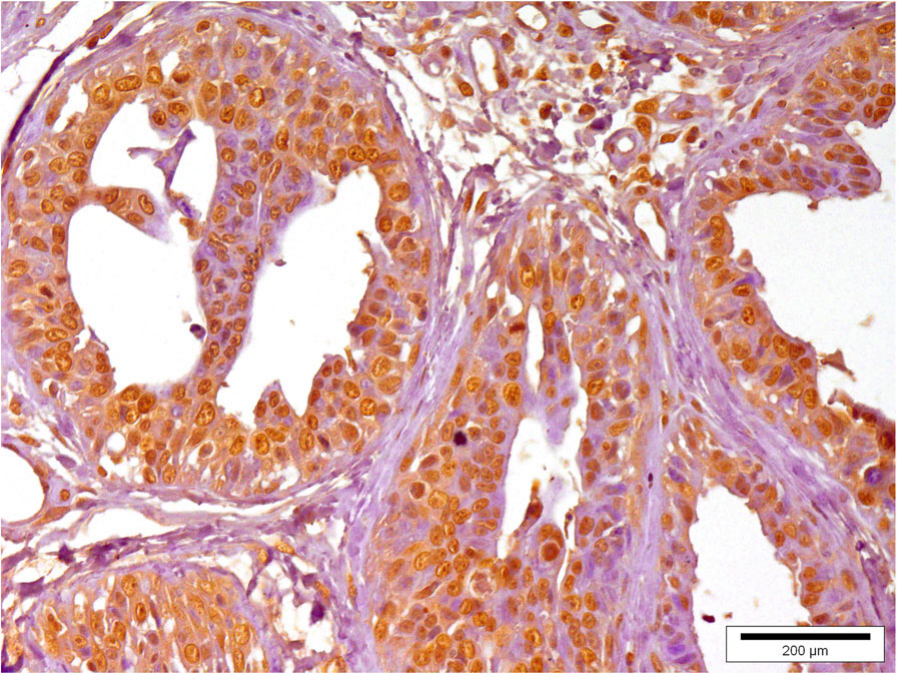
Immunohistochemistry of canine mammary tumor (Simple carcinoma showing mammaglobin-B immunopositive cells). Bar = 200 μm

**Fig. 6:**
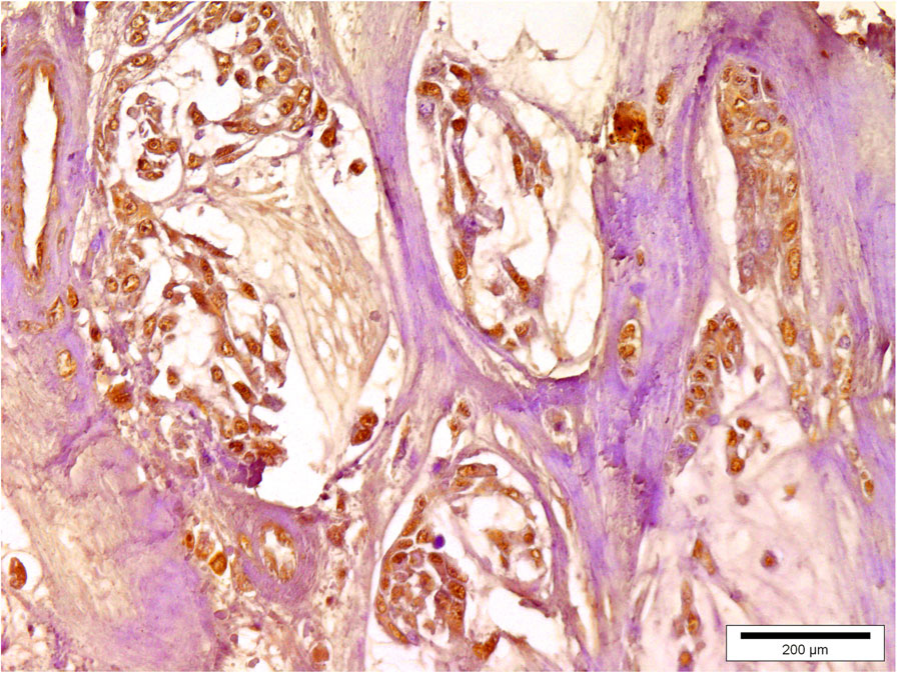
Immunohistochemistry of canine mammary tumor (Carcinosarcoma showing mammaglobin-B immunopositive cells). Bar = 200 μm

**Fig. 7:**
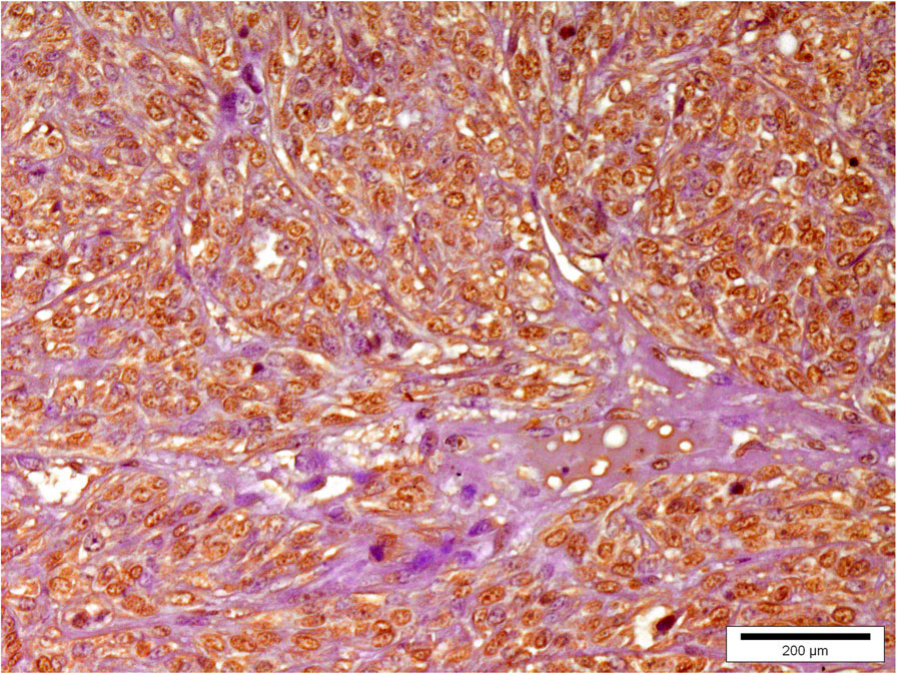
Immunohistochemistry of canine mammary tumor (Fibrosarcoma showing mammaglobin-B immunopositive cells. Bar = 200 μm

A low mammaglobin-B protein expression was observed in healthy mammary tissue (Fig. 3). Only 6.67% of healthy mammary tissue sections showed SI ≥6. However, mammaglobin-B specific immunostaining was observed in all the CMT sections with a clear variation in the percentage of immuno-positive cells. A strong positive immunoreactivity to the protein was detected in most of the CMT tissues (Figs. 4, 5, 6, 7) indicating higher expression of mammaglobin-B protein in tumor cells. Thus, a higher level of immunostaining was seen in 23 out of 30 specimens (76.7%). All the complex carcinomas showed a higher level of mammaglobin-B expression (i.e. SI score ≥ 6), whereas, for simple carcinomas higher protein expression was observed in 63.63% cases. For carcinosarcoma and fibrosarcoma, 50% of the cases had higher expression of mammaglobin-B protein with SI score ≥ 6. Moreover, 70% of grade I, 83.33% of grade II and 50% of grade III CMT cases had higher mammaglobin-B protein expression with SI score ≥ 6.

### Correlation between mammaglobin-B mRNA and protein expression in different histotypes and grades of CMT

**Fig. 8:**
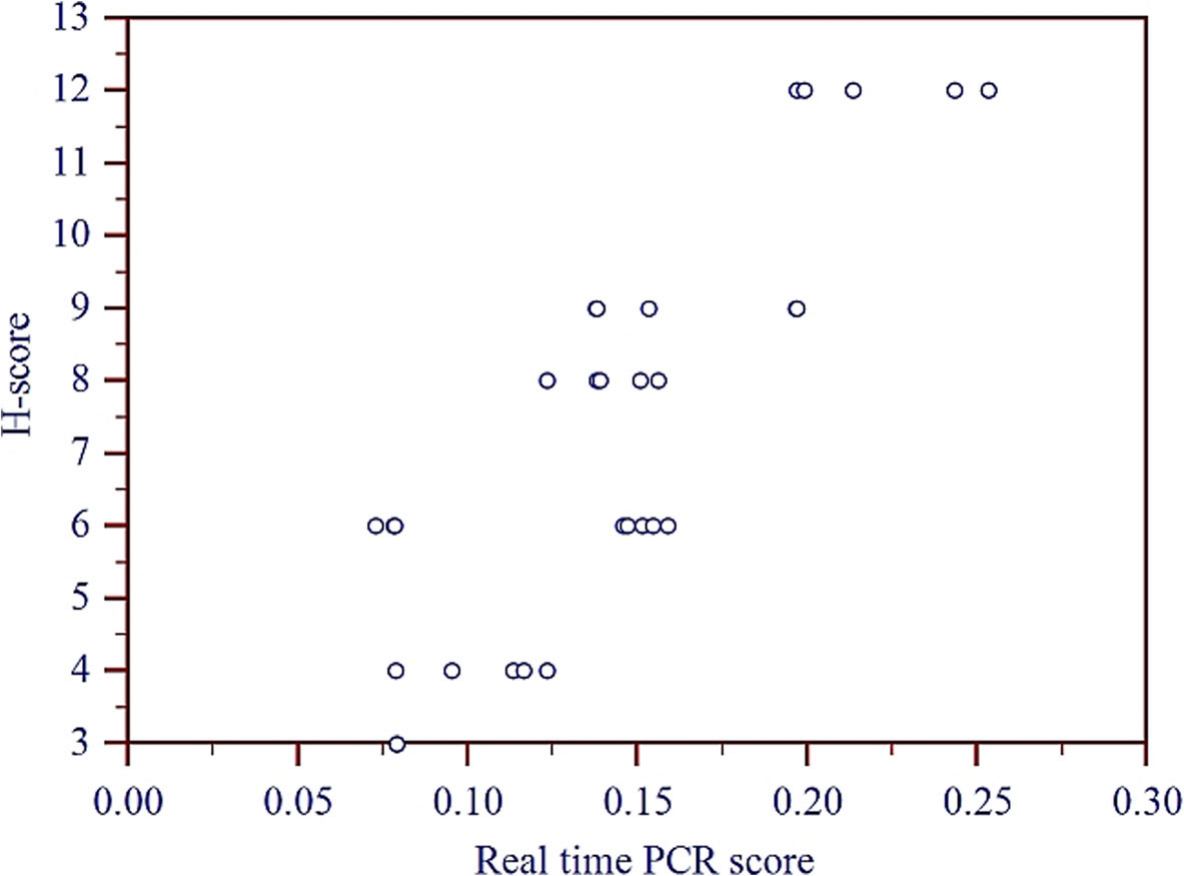
Scatter plot showing positive correlation between mammaglobin-B transcript and protein expression

Scatter plot, demonstrated a positive and significantly high correlation (*P* < 0.0001) between mammaglobin-B transcript and protein expression (Pearson Correlation coefficient = 0.8394) (Fig. 8). Spearman’s rank correlation between H-score and 2^−ΔCT^ was found to be 0.762 (*P* < 0.0001) indicating that the rankings for mammaglobin-B transcript and protein expression among different specimens were highly similar. A regression  equation (H = 0.357 + 48.335X; where H represents H - score and X represents the qRT-PCR score) was obtained which could predict H-score from the qRT-PCR score with r^2^ value of 70.47%. The relationship between both the scores was found to be linear, suggesting that mammaglobin-B transcript appropriately predicts the protein expression in CMT. However, the Kruscal Wallis test revealed no significant differences in terms of H score and qRT-PCR score with respect to CMT histotypes or tumor grades, indicating that mammaglobin-B over-expression occured irrespective of the histological parameters studied.

## Discussion

Dogs, primarily unspayed bitches are most frequently affected by mammary neoplasia which are usually diagnosed at a late end point of carcinogenesis making them irresponsive to treatment [1]. One of the best ways to increase efficacy of CMT treatment is by diagnosing them in their early stages. This can be achieved only by employing those biomarkers which are capable enough to distinguish the tumorous glands from the healthy ones. The present panel of canine mammary tumor biomarkers are mostly non-specific that fail to aid early tumor detection. In the past, we have put considerable efforts in search of biomarkers for the diagnosis and prognosis of CMT [18, 30]. So, in the line of thought to explore out a best suited panel of biomarkers for diagnosing mammary tumors we studied the mammaglobin-B gene, because a single biomarker is never 100% efficient to give a perfect conclusion. Specificity and sensitivity of mammaglobin to detect human breast cancer makes it a promising biomarker [31]. Till now, to our best knowledge there is only one report from our laboratory in which we had investigated the potential of mammaglobin-B protein as a serum biomarker of canine mammary carcinomas [14]. However no information is available on its mRNA expression levels in CMT. So in this study, we evaluated the expression of mammaglobin-B mRNA in CMT by qRT-PCR. The gene was found to be significantly up-regulated (6.663 ± 0.841 times) in CMT compared to the healthy glands. Our findings corroborate with the earlier studies carried out on human breast cancers which showed that mammaglobin transcript levels are higher by 10 folds in comparison to healthy breast tissue [3, 32].

Histopathology and IHC examination has been playing an important role as a diagnostic tool for neoplasms and pathogens [32, 33]. In human breast pathology, IHC is routinely used in the diagnosis and prognosis of breast neoplasia [34, 35]. In the present investigation, we found that majority of the examined CMT sections were carcinomas in nature, similar to the report of breast cancer [36]. According to Suchy et al., (2000) [37] the mammaglobin expression in breast tumors did not show any correlation with histological type, tumor grade, tumor stage or hormone receptor status. In corroboration with their findings, we also did not find any significant differences in terms of H score and qRT-PCR score with respect to CMT histotypes or their grades, suggesting that mammaglobin-B over-expression was irrespective of CMT histological types or grades. Overall, the protein was highly expressed in 76.7% of CMT tissues. However, in most of the healthy canine mammary tissues, a lower level of protein expression (SI score < 6) was observed. Our findings are in accordance with the earlier reports, where a high frequency (~ 80%) of mammaglobin protein expression was observed specifically in human breast cancer tissue [38, 39]. In the present study, all the complex mammary carcinoma sections had higher mammaglobin-B expression as evidenced by their higher SI score. Whereas for simple carcinoma, mammaglobin-B expression was higher in 63.3% positive cases in the category. While, 50% each of carcinosarcoma and fibrosarcoma sections had high mammaglobin-B expression. The expression pattern of this protein in CMT types was different when compared to similar human breast cancer counterparts studied in the past [40, 41]. The likely reason for this difference in expression may be the use of antibody specific to canine mammaglobin-B protein. Moreover, in the current study, no significant differences in terms of H score and qRT-PCR score with respect to CMT histotypes or tumor grades were observed which implies that, mammaglobin-B is overexpressed irrespective of histological types or grades of CMT. So, this protein should be considered important while identifying any tumor of canine mammary gland origin using immunohistochemical staining.

## Conclusion

Our study is the first ever attempt made to report overexpression of mammaglobin-B at mRNA and protein levels in CMTs. Here we also report a significant correlation between qRT-PCR and IHC scores for the diagnosis of mammary gland neoplasia in canines. Our results may provide the basis for developing a novel approach in veterinary diagnostic research, although additional studies using a large number of healthy and pathological clinical samples are required to validate these preliminary findings. Further, it would also be interesting to correlate the mammaglobin-B expression with other clinico-pathological parameters of CMT in future. As mammaglobin-B is highly expressed in CMT and to a low degree in healthy mammary tissues, it is capable of discriminating CMT subjects from healthy controls. Adding this protein to the current panel of diagnostic biomarkers will definitely improve the sensitivity and specificity of CMT diagnosis.

## Abbreviations

cDNA: Complementary DNA
CMT: Canine mammary tumor
DAB: 3,3′-Diaminobenzidine
HE stain: Haematoxylin and eosin stain
HRPO: Horse radish peroxidase
IHC: Immunohistochemistry
NBF: Neutral buffered formalin
qRT-PCR: Quantitative real time polymerase chain reaction
RPS 19: Ribosomal protein S19
SAS: Statistical analysis software
β-actin: Beta actin
